# Exploring the Cancer Therapeutic Potential of Traditional Medicinal Plants by Modulating the Apoptotic Pathway

**DOI:** 10.1155/bmri/2086738

**Published:** 2026-04-24

**Authors:** Mithila Kulkarni, Mohammad Amjad Hussain, Reginald Samson Valdar, Shreya Hebbar, Suparna Laha

**Affiliations:** ^1^ Cell Biology and Molecular Genetics Division, Yenepoya Research Centre, Yenepoya (Deemed To Be University), Mangalore, Karnataka, India; ^2^ Specialized Research Unit 4, Yenepoya Medical College, Yenepoya (Deemed To Be University), Mangalore, Karnataka, India, yenepoya.edu.in

**Keywords:** apoptosis, BAX/BCL-2, cancer severity, genomic instability, phytochemicals

## Abstract

**Introduction:**

Cancer is a versatile disease with a high rate of relapse and mortality. Despite the presence of various treatment modalities, there is a serious need for the development of its management. Phytochemicals, which are abundant in nature, can be turned into novel medications for these types of diseases by optimizing their functions and structures. Therefore, a thorough understanding of traditional medicine and medicinal plants may lead to the discovery of new and affordable medicines. Many studies have shown that a variety of medicinal plants and nutraceuticals derived from different natural resources, along with their byproducts, such as flavones, flavonoids, antioxidants, and major polyphenolic constituents, offer significant protection against a wide range of cancers. These dietary compounds may function as chemopreventive agents, as they have been shown to possess an inhibitory effect on cancer cells.

**Methodology:**

A comprehensive literature review was conducted to identify medicinal plants traditionally used for their anticancer properties. Studies presenting molecular evidence for the mechanisms of action of these plants were critically analyzed and selected for inclusion.

**Results:**

Our analysis of the available research data on ethnic medicinal plants revealed that they affect selective pathways, such as the cell cycle, proliferation, apoptosis, metastasis, angiogenesis, and phagocytosis, leading to tumor‐static and cell death of cancer cells.

**Conclusion:**

A wide range of traditional plants have been utilized for the treatment of different diseases, including cancer. Despite the presence of various anticancer medicinal plants, only a few have advanced beyond the laboratory and are used clinically. This is primarily due to the lack of knowledge regarding their mode of action at the molecular level. By understanding the molecular mechanisms, these traditionally used medicines can be translated into drugs and used to manage diseases such as cancer.

## 1. Introduction to Medicinal Plants

Cancer remains a dreadful disease and is one of the leading causes of morbidity and mortality worldwide. Recent global estimates indicate that there are approximately 20 million new cancer cases and around 10 million cancer deaths each year, accounting for about one in six deaths globally. The annual number of new cases is projected to rise substantially in the coming decades owing to population growth, ageing, and the increasing prevalence of risk factors such as tobacco use, obesity, unhealthy diets, and environmental pollution. These trends highlight an urgent need for more effective and accessible strategies for cancer prevention and treatment (WHO). Current cancer management relies on a combination of surgery, chemotherapy, radiotherapy, targeted therapies, and immunotherapies. Although these modalities have significantly improved survival in several malignancies, they have important limitations. Chemotherapy and radiotherapy are constrained by systemic toxicity, narrow therapeutic windows, and damage to normal tissues, which limit dosing and negatively affect quality of life, whereas intrinsic and acquired drug resistance frequently leads to relapse and treatment failure. Targeted therapies and immunotherapies, despite producing remarkable responses in selected patients, are associated with heterogeneous response rates, immune‐related adverse events, emergence of resistance, high cost, and limited availability in many low‐ and middle‐income regions. Together, these lacunae underscore the need for complementary and alternative anticancer strategies that can selectively target tumor cells, overcome resistance, and ideally exhibit reduced systemic toxicity [1]. Phytochemicals and pharmacologically active secondary metabolites detected in medicinal plants are abundant and can be converted into novel medications for these types of diseases by optimizing their functions and structures [2]. The chemical composition of medicinal plants includes a range of bioactive metabolites, such as alkaloids, tannins, terpenoids, saponins, flavonoids, and phenols [3]. Antioxidants, neuropharmacological agents, immunity boosters, detoxifying agents, and anticancer agents are the primary functional classes of phytochemicals with therapeutic potential [4]. Medicinal plants are essential for human health and well‐being and represent an important source of cures and remedies due to the presence of phytochemicals and metabolites. Currently, 80% of the global population relies on plant‐based medications. In addition, many medications have been derived from medicinal plants. Medications for therapeutic purposes have long been derived from these natural chemicals, including quinine (found in *Cinchona*), ergotamine (found in infected rye), salicylates (found in willow bark), and digitalis (found in foxglove). Natural drug development is a complex process that combines biological, phytochemical, and molecular methods. Therefore, medicinal plants are often used as pharmaceutical raw materials and home cures [5]. However, the quality, safety, and efficacy standards of medicinal herbs may not be met [6]. Therefore, a thorough understanding of traditional medicine and medicinal plants, as well as the scientific study of their chemical components and mode of action, may lead to the discovery of new and affordable medicines.

In this review, we first provide an overview of medicinal plants and their phytochemicals in the context of cancer, highlighting key classes of bioactive compounds and their general roles in carcinogenesis and chemoprevention. We then focus on a subset of traditional medicinal plants for which experimental evidence supports anticancer activity and at least one apoptosis‐related mechanism of action, describing how these plants modulate specific molecular pathways across different cancer types. Finally, we discuss safety and translational considerations, including toxicity, chemotype variability, extract standardization, and the limitations of current preclinical data to outline both the opportunities and the challenges in repurposing traditional medicinal plants as clinically useful anticancer agents.

## 2. Medicinal Plants and Cancer

Medicinal plants are typically found in specific and occasionally harsh habitats, such as mountains, deserts, and tropical rainforests, which contribute to the development of their unique qualities [7]. They also play an essential role in the heterologous expression of valuable medicinal compounds in plants. Knowledge of several genes encoding biosynthetic pathways in medicinal plants is now available owing to current omics techniques and traditional biochemical methods. With the use of recombinant technology, these genes and regulatory elements are introduced into bacteria and yeast, which act as host species, leading to the heterologous production of secondary metabolites, such as terpenoids [8]. Some metabolites, such as the mitostatic medication paclitaxel (Taxol), restrict cell division and are therefore used to treat cancer. In recent years, genetic engineering has enabled the development of cancer‐targeting medications from plants, particularly through gene editing methods such as the CRISPR/Cas9 platform [9]. Furthermore, the production of various novel proteins due to the temporary expression of foreign recombinant proteins in plants is of pharmaceutical importance. Therefore, plants can be used as tools for the production of vaccines, growth regulators, human blood products, and antibodies [8].

**Figure 1:**
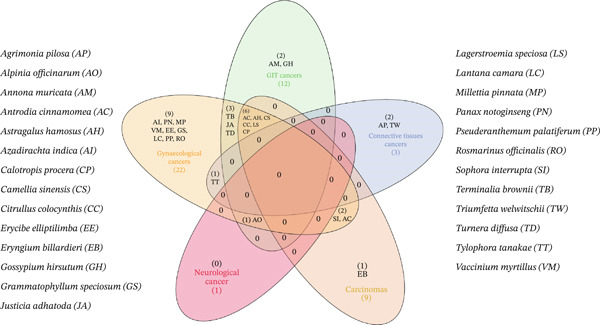
Venn diagram showing the overlap between medicinal plants and cancer types. Shared regions represent plants reported to act against multiple cancers, whereas nonoverlapping areas indicate plants specific to a single cancer type. Inside the Venn diagram, abbreviations are mentioned, and on the left‐ and right‐hand sides, the full form has been provided.

Many studies involving animal models and cell culture systems have shown that a variety of medicinal plants and nutraceuticals derived from different natural resources, along with their byproducts, such as flavones, flavonoids, antioxidants, and major polyphenolic constituents, offer significant protection against a wide range of cancer types (Table 1, Figure 1). These dietary compounds, including baicalein, biochanin A, and fisetin, may function as chemopreventive agents as they have demonstrated inhibitory effects on cancer cells. Terpenoids are one of the most diverse and abundant classes of plant‐derived compounds and are extensively used in multiple industries, including pharmaceuticals and food. A wide range of terpenoid derivatives has been isolated from plants, highlighting their biosynthetic diversity and functional specificity [13]. The chemopreventive and anticancer mechanisms of these natural substances are discussed in this review.

**Table 1:** Compiled research work on traditional plants revealing their mode of action.

Division	Cancer	Scientific name	Cell line/animal involved	Extract	Dose/concentration	End result	Markers
Gynecological cancers	Breast	*Sophora interrupta*	MCF7	Ethyl acetate	250 *μ*g/mL	Necrosis factor‐related apoptosis	TRAIL/DDX3
		*Terminalia brownii*	MCF7	Ethyl acetate	20 *μ*g/mL	Apoptosis	S phase cell cycle
				Chloroform	25 *μ*g/mL		
				Hexane	25 *μ*g/mL		
		*Vaccinium myrtillus*	MCF7	Crude extract	0.3–0.4 mg/mL	Apoptosis	Arrest of the G2/M phase
		*Justicia adhatoda*	MCF7	Methanolic	161.57 *μ*g/mL	Apoptosis	BAX and Caspase‐3 upregulation, BCL‐2 downregulation
		*Alpinia officinarum*	MCF7	Methanolic	0–100 *μ*g/mL	Apoptosis and antiproliferative	Cyclin A, E2F1, and CDK2 reduction
		*Antrodia cinnamomea*	MCF7	Ethanolic	185.043 *μ*g/mL	Apoptosis	Activation of skp2/microRNAs pathway
		*Erycibe elliptilimba*	SKBR3, MDA‐MB 435	Methanolic	56.07 *μ*g/mL	Antiproliferative activity	Arrest at the G2/M phase
					30.61 *μ*g/mL		
		*Annona muricata*	BT20, MCF7	Ethyl acetate	1–100 *μ*g/mL	Decrease cell viability	NF‐*κ*B and p65 protein expression
		*Astragalus hamosus*	MCF7	Chloroform	253.2 *μ*g/mL	Apoptosis	Down regulation of BCL‐2 and Ki67 and upregulation of caspase‐3, 8, and 9 genes
		*Grammatophyllum speciosum* pseudobulb	MCF7	Ethanol	0–1000 *μ*g/mL	Antiproliferative activity	Reduce AKT/*β*‐catenin pathway activity
		*Lagerstroemia speciosa*	MCF7		5–50 *μ*M	Antiproliferative activity	Reduced pJAK2 and STAT3 proteins
		*Lantana camara*	MCF7	Ethanolic	46.63 *μ*g/mL	Apoptosis	Increased BID, BAX, and a decrease in BCL‐2.
		*Turnera diffusa*	T47D	Methanolic	54.02 *μ*g/mL	Apoptosis	Caspase 3 pathway
		*Millettia pinnata*	MCF‐7	Methanolic	4.4 *μ*M	Antiproliferative activity	Cell cycle arrest at the G0–G1 phase
		*Camellia sinensis*	MCF‐7	Ethyl acetate	23.45 *μ*g/mL	Apoptosis	Caspase activation [10]
		*Calotropis procera*	MCF‐7	Chloroform	135 *μ*g/mL	Antiproliferative, antiangiogenic properties	Induction of ROS activity
		*Citrullus colocynthis*	MCF‐7	Methanolic	30 *μ*g/ml	Inhibit proliferation	Upregulation of p21/p27 followed by downregulation of cyclin A/E and CDK2
	TNBC	*T. diffusa*	MDA‐MB 231	Methanolic	30.67 *μ*g/mL	Apoptosis	Caspase 3 pathway
		*Pseuderanthemum palatiferum*	MDA‐MB 231	Ethanolic	100–500 *μ*g/mL	Cytotoxic activity and apoptosis	
		*V. myrtillus*	MDA‐MB 231	Ethyl acetate	30 *μ*g/mL	Apoptosis	Arrest of the G2/M phase
		*C. sinensis*	MDAMB‐231		5–10 *μ*M	Inhibiting cell proliferation	Cell cycle arrest
		*L. speciosa*	MDAMB‐231		5–50 *μ*M	Antiproliferative activity	Reduced pJAK2 and STAT3 proteins
		*L. camara*	MDAMB‐231	Ethanolic	40–150 *μ*g/mL	Increase in BID and BAX and a decrease in BCL‐2.	Increased BAX and decreased BCL‐2.
		*T. brownii*	MDAMB‐231	Crude extract	115 *μ*g/mL	Apoptosis	Caspase‐3
		*A. hamosus*	MDAMB‐231		817 *μ*g/mL	Apoptosis	Down regulation of BCL‐2 and Ki67 and upregulation of Caspase‐3, 8, and 9 genes
	Cervical	*T. diffusa*	SiHa, C‐33	Methanolic	50.14 *μ*g/mL	Apoptosis	Caspase 3 pathway
		*Azadirachta indica*	HeLa	Methanolic	115 *μ*g/mL	Apoptosis	Upregulation of Caspase 3
		*Panax notoginseng*	HeLa	DMSO	10.27 *μ*g/mL	Apoptosis	Increased expression of BAX, Caspase‐3, 8, 9
		*Tylophora tanakae*	KB3 1, KBVI		7 nM		
	Ovary	*Rosmarinus officinalis*	A2780		2.5–20 *μ*g/mL	Apoptosis	Downregulation of BCL‐2
GI track cancers (esophagus, stomach, small and large intestines, rectum, anus, liver, pancreas, and biliary system)	Colon	*T. brownii*	HCT 116	Ethyl acetate	15 *μ*g/mL	Apoptosis	Caspase‐3
				Chloroform	19 *μ*g/mL		
				Hexane	20 *μ*g/mL		
		*A. muricata*	HT29, HCT116	Ethyl acetate	11.43 *μ*g/mL	Apoptosis	Downregulation of antiapoptotic BCL‐2 protein
					8.98 *μ*g/mL		
		*Gossypium hirsutum*	COLO 205		3.2 *μ*M	Apoptosis	Reduced the expression of the BCL‐2
		*J. adhatoda*	HT29	Cyclohexane‐ ethyl acetate	1 *μ*M	Apoptosis	Caspase‐3, BAX, and cleaved‐PARP
		*C. sinensis*	HT29		0–50 *μ*M	Decreased angiogenesis	Downregulating matrix metalloproteinase 9 (MMP‐9)
	Gastric	*A. officinarum*	MGC 803		18.685 *μ*M	Apoptosis	Decreased BCL‐2 and increased levels of cleaved Caspase‐3 and cleaved PARP.
	Liver	*A. officinarum*	HCCL‐3,Hu‐7	Liquid chromatography‐mass spectrometry/mass spectrometry‐based extraction	40, 80, and 120 *μ*M	Apoptosis	Decreased BCL‐2 and increased levels of cleaved Caspase‐3 and cleaved PARP.
		*T. brownii*	HEPG‐2	Ethyl acetate	11.6 *μ*g/mL	Apoptosis	
				Chloroform	12.3 *μ*g/mL		
				Hexane	15 *μ*g/ml		
		*T. diffusa*	Hep G‐2	Methanolic	43.87 *μ*g/mL	Apoptosis	Caspase 3 pathway
		*L. speciosa*	Hep G‐2	Ethanolic	25–150 *μ*g	Apoptosis	Activation of Caspase‐8, 9, and 3
		*C. procera*	HEP‐ G2			Antiproliferation	Lowering of Cenpf mRNA

Connective tissue cancers	Fibrosarcoma	*Agrimonia Pilosa*	HT1080	Methanolic	10–20 *μ*g/mL	Antiproliferation	Decreasedphospho‐ERK/JNK, AKT‐1
	Leukemia	*Triumfetta welwitschii*	Jurkat	Methanolic	19 *μ*g/mL	Apoptosis	DNA fragmentation
		*T. tanakae*	MT‐1,MT‐2	Methanolic	0.05–100 *μ*g/mL	Antiproliferative activity	NF‐*κ*B pathway inhibition
		*C. colocynthis*	A375	Methanolic	400–500 *μ*g/mL	Apoptosis	Upregulating the levels of BAX and decreasing BCL‐2

Carcinomas (tumors that form on the skin or in the lungs, breasts, prostate, colon, kidneys, pancreas, etc.	Lung	*C. colocynthis*	A549	Ethanolic	42 *μ*g	Apoptosis	Decrease BCL‐2
		*A. cinnamomea*	A549	Crude	101 *μ*g/mL	Apoptosis	Induces S‐phase arrest
		*A. hamosus*	A549	Water soluble extract	1–50 *μ*M	Apoptosis	Down regulation of BCL‐2 and Ki67 and upregulation of Caspase‐3, 8, and 9 genes
		*L. speciosa*	A549	Ethanloic	841.23 *μ*g/mL	Apoptosis	Activation of Caspase‐8, 9, and 3
		*A. officinarum*	A549		20 *μ*M	Apoptosis	Decreased BCL‐2 and increased levels of cleaved Caspase‐3 and cleaved PARP.
		*C. procera*	H1299	Ethanolic	0–15 *μ*g/mL	Apoptosis	ROS generation
	Prostate	*C. procera*	PC3	Methanolic	5.1 *μ*g/mL	Apoptosis	Downregulation of ERK1/2 activation [11]
		*S. interrupta*	PC‐3	Ethanolic extract	700 *μ*g/mL	Apoptosis and Wnt‐*β*‐catenin signaling mediated cancer cell death	DDX3
		*A. muricata*	Capan‐1	Hexane	8 *μ*g/mL	Decreases cell viability	BAX‐BAK/Caspase‐3 activation
		*Astragalus myrtillus*	LNCaPDU‐145	Methanolic	40.78 *μ*g/mL	Apoptosis	Down regulation of BCL‐2 and Ki67 and upregulation of Caspase‐3, 8, and 9 genes
		*C. sinensis*	PC‐3	Water	600 *μ*M	Decreased cell proliferation	G2/M cell cycle arrest [12]
	Kidney	*L. speciosa*	Caki		2.5–10 *μ*M	Apoptosis	Activation of Caspase‐8, 9, and 3
	Pancreas	*Eryngium billardieri*	PANC‐1	n‐hexane	50–200 *μ*g/mL	Apoptosis	Increased expression of BAX
				Dichloromethane	50–200 *μ*g/mL		
				Methanolic	50–200 *μ*g/mL		

	Neuroblastoma	*A. officinarum*	In vivo		3.75 *μ*M	Apoptosis	Upregulation of Caspase‐3

## 3. The Traditionally Used Medicinal Plants and Their Mode of Action as Anticancer Drugs

**Figure 2:**
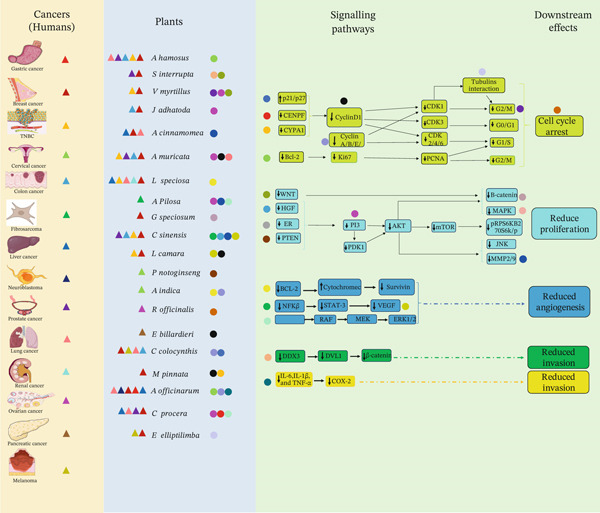
Graphical representation showing the interactions between different cancer types, medicinal plants, and signaling pathways. Each colored circle represents the connection between a plant and the signaling pathway it regulates, whereas each colored triangle represents the link between specific cancers (or the affected organ) and the plants implicated in their modulation. Together, the diagram highlights how plant‐derived compounds influence key oncogenic pathways across multiple cancer types.

**Figure 3:**
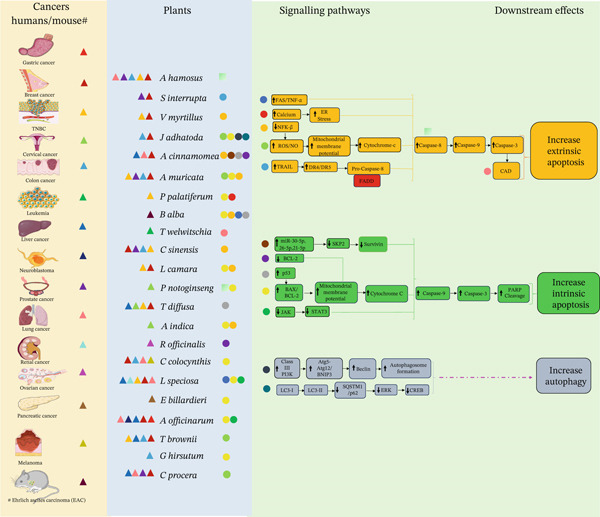
Graphical representation showing the interactions between different cancer types, medicinal plants, and signaling pathways. Each colored circle represents the connection between a plant and the signaling pathway it regulates, whereas each colored triangle represents the link between specific cancers (or the affected organ) and the plants implicated in their modulation. Together, the diagram highlights how plant‐derived compounds influence key oncogenic pathways across multiple cancer types.

A wide range of traditional plants have been utilized for the treatment of different diseases associated with the digestive system, respiratory disorders, kidneys, blood, liver, oxidative stress, and inflammation, and have antimicrobial activity. Despite the presence of various anticancer medicinal plants, only a few have advanced beyond the laboratory and are used clinically. This is primarily due to the lack of knowledge of the mode of action at the molecular level [14]. In this review, we have focused on only a few traditional plants that show anticancer properties, and preliminary research on their mode of action has been conducted. We analyzed the mode of action of these plants and their crosstalk, which will help open up new avenues to repurpose these traditional medicines for cancer therapy (Figures 2 and 3).

### 3.1. Globally Recognized Medicinal Plants

Certain plants are found worldwide and have a strong background of use as traditional medicine in various cultures.

#### 3.1.1. *Calotropis procera*

*C. procera* commonly referred to as milkweed, is a perennial shrub within the Apocynaceae family and the Asclepiadoideae subfamily. It is native to Africa, Western Asia, the Arabian Peninsula, Indo‐China, and the Indian Subcontinent. It has also been naturalized in various parts of Australia and the United States [15]. This plant is also widely used in traditional medicine throughout the Middle East, North Africa, and Southeast Asia to treat different health conditions, including respiratory ailments (asthma and colds), skin‐related disorders (eczema), digestive issues (indigestion and diarrhea), joint‐related issues (rheumatism), and infections such as dysentery, elephantiasis, and leprosy. Extracts obtained from the aerial sections of plants, particularly decoctions, are frequently used to manage muscle spasms, alleviate fever, address constipation, and relieve joint pain. The therapeutic potential of this plant is attributed to the presence of various secondary metabolites and cardiotonic compounds [15]. Phytochemical analysis has revealed the presence of bioactive compounds such as cardenolides, oxypregnanes, triterpenoids, flavonoids, sterols, and glycosides [16]. Several compounds have been found to affect ER+ breast cancer cells at a drug concentration of 135 *μ*g/mL, reducing proliferation through mechanisms, including cytotoxicity, apoptosis, and autophagy. Moreover, oxypregnanes and oligoglycosides exhibit cytotoxic activity against prostate cancer cells with the dose concentration ranging from 5.1 *μ*g/mL and glioblastoma cells [15]. In vitro analysis has revealed that the phenolic extract of *C. procera* exhibits anticancer activity by reducing cell viability through the activation of apoptosis, scavenging of reactive oxygen species (ROS), and downregulating the activation of proteins involved in cell motility. This extract also downregulated the activation of PI3K/AKT/mTOR proteins involved in the pathway, suggesting that it is the primary target of *C. procera*. In vivo analysis of this plant extract also revealed a reduction in the activation of the ERK1/2 pathway in tumor tissues, along with a reduction in angiogenesis within tumor and lung tissues. Moreover, the reduction in Cenpf mRNA levels in lung with dose concentration of 1–5 *μ*g/mL represents the anti‐invasive cancer potential of *C. procera* [17].

#### 3.1.2. *Citrullus colocynthis*

*C. colocynthis* referred to as colocynth, is a prominent medicinal plant and source of oil, with archeological evidence of its seeds dating back to 4000 BC [18]. This plant has been commonly employed in traditional medicine, especially in subtropical and tropical regions, to treat multiple diseases, such as asthma, diabetes, bronchitis, cancer, and jaundice [19, 20]. Genetic analysis of this plant has demonstrated clear differentiation according to geographical location, with distinct haplotypes and variations in nucleotide substitution rates. India and Pakistan exhibit the highest levels of nucleotide substitutions, whereas the Middle East and West Asia show the lowest [20]. This genetic diversity indicates its adaptability and distribution in desert environments [19]. As a perennial herb of the Cucurbitaceae family, *C. colocynthis* exhibits diverse pharmacological properties that have been substantiated in contemporary phytotherapy [20]. The fruit and seed extracts of this plant have demonstrated anticancer and antidiabetic activities. In addition, since these fruits contain cucurbitacin, which has served as the basis for the development of novel anticancer and antitumor agents, they exert their effects by inhibiting tumor growth by modifying cell physiology. It was also observed that methanolic extracts of this plant suppressed breast cancer cells by inducing cell cycle arrest via upregulation of p21/p27, followed by downregulation of cyclin A/E and CDK2 mRNA levels at a dose of 30 *μ*g/mL, as confirmed by q‐PCR, and prevented further proliferation of cancer cells [21]. The fruit extract of this plant has also shown cytotoxic effect in melanoma cells by regulating the levels of Bax and BCL‐2 (downregulation of BCL‐2 and upregulation of Bax‐2) genes (at 400–500 *μ*g/mL dose concentration), confirmed by q‐PCR, resulting in cell apoptosis and reduced cell proliferation [22]. Furthermore, the presence of antioxidants, such as flavonoids, contributes to inhibiting the growth of colon cancer cells, highlighting the plant′s therapeutic potential [23]. Ethanolic extracts and cucurbitacins B, E, and E‐glucopyranoside also show cytotoxicity and immunomodulatory effects on PBMCs and lung cancer cells at 1–50 *μ*M concentration [23, 24]. The fruit extract of this plant has also shown cytotoxic effects in melanoma cells by regulating the levels of Bax and BCL‐2 (downregulation of BCL‐2 and upregulation of Bax‐2), resulting in cell apoptosis and reduced cell proliferation [24].

#### 3.1.3. *Sophora interrupta*

*S. interrupta* commonly known as Adavi Billa in Ayurveda, is a shrub that is identified as part of the Fabaceae family. This plant is distributed across tropical and temperate regions worldwide, particularly in the Eastern Ghats of Andhra Pradesh, India. It has been used in traditional remedies for its antioxidant, anti‐inflammatory, antiproliferative, and anticancer activities [25]. *S. interrupta* has been studied in prostate adenocarcinoma and ER^+^ breast cancer to evaluate its potential as an anticancer agent. *S. interrupta* root ethyl acetate (SEA) extract induces structural alterations, including membrane blebbing, loss of cell adhesion, and rounded cellular shape in breast cancer cells compared with normal cells at an IC50 value of 250 and 700 *μ*g/mL in ER+ breast cancer and prostate adenocarcinoma, respectively. The root extract exhibited antiangiogenic, antioxidative, and anti‐inflammatory activities. This markedly inhibited angiogenesis. Research indicates that the secondary metabolite of *S. interrupta* establishes six hydrogen bonds with the DDX3 protein at amino acid residues Arg 202, Gln 207, Gly 227, Gly 229, Thr 231, and Ala 232. This interaction suggests a role in modulating the cell cycle, triggering apoptosis, and promoting cancer cell death through the Wnt‐*β*‐catenin signaling pathway [25]. The chemicals found in this plant may also help in TRAIL‐mediated cell death [25]. In addition, these metabolites established strong hydrogen bonds with Lys 920, Thr 916, Cys 919 (two bonds), and Glu 917 of VEGFR2, resembling the binding pattern of the antiangiogenic drug axitinib [26].

### 3.2. Traditional Plants of the Asian Continent

Our search for traditional plants with mechanistic studies on anticancer properties revealed that a large number of medicinal plants from Asia are taken up for extensive research in order to find their mode of action against cancer. The following section highlights the research findings on the mechanisms of action of these traditional plants of the Asian continent (Figures 2 and 3).

#### 3.2.1. *Agrimonia pilosa*

*A. pilosa* is commonly referred to as desiccated grass. It is also known by several other common names, such as melon herb and dragon′s tooth grass, and belongs to the Rosaceae family. It is native to China, Japan, and Korea. It has been widely used for edible purposes for many years. Traditionally, *A. pilosa* has been used in herbal medicine because of its healing properties. It has been used to control bleeding, treat malaria and dysentery, aid detoxification, and serve as a booster for blood deficiency. It has also been used to treat hemoptysis, hematemesis, carbuncles, sores, bloody dysentery, itching, dehydration, and strain. Recent phytochemical analysis revealed that the plant contains approximately 252 phytochemicals, mostly consisting of flavonoids, isocoumarins, volatile oils, pentacyclic triterpenoids, tannins, m‐benzotrienols, phenols, organic acids, and lignans. These phytochemicals demonstrate various medicinal traits, including anticoagulant, antioxidant, anticancer, sedative, anti‐inflammatory, gastrointestinal protective, and antifatigue effects. Due to its various pharmacological activities, it is also used in the drug industry [27]. Recent studies have revealed the use of *A. pilosa* for the effective treatment of fibrosarcoma, a rare cancerous tumor that develops in the fibrous connective tissue of the body. Immunofluorescence investigation has demonstrated that there is reduced expression of matrix metalloproteinase (MMP) protein, too, in a dose‐related manner due to the methanolic extract of *A. pilosa.* This extract also downregulated the protein expression of p‐ERK, p‐JNK, and AKT1 with drug concentration ranging from 10 to 20 *μ*g/mL. This inactivation of ERK and JNK can prevent the invasion of tumor cells. Furthermore, this extract decreased the activation and expression levels of other proteins like matrix metalloproteinase 9 (MMP‐9), MMP‐2, and, in addition to AKT1, as confirmed by western blotting. As a result, *APL* may be a useful medication for preventing metastasis by downregulating ERK, JNK, and MMP proteins [28].

#### 3.2.2. *Alpinia officinarum*

*A. officinarum* also called lesser galangal, belongs to the family Zingiberaceae. The species variation of this plant is significant, with its origin in China. It is distributed across several provinces, including Fujian, Taiwan, Guangdong, Guangxi, and Hainan [29]. It is cultivated in various Asian countries, including Egypt, India, Sri Lanka, Thailand, and Malaysia [30]. For decades, this plant has been traditionally utilized in the form of decoction, juice, or infusion, alone or in combination with other herbs, food, or beverages, to manage a range of conditions such as the common cold, inflammation, and stomach‐related pains. It demonstrates multiple pharmacological actions, such as anti‐inflammatory, antiemetic, antioxidant, cytotoxic, and antimicrobial properties [31]. Several bioactive compounds extracted from the rhizome of this plant exhibit anti‐inflammatory and antioxidant properties due to the presence of phenolic compounds, including 2‐DAH (2‐diarylheptanoids), isorhamnetin, KFD (kaempferide), galangin, and 5‐HHMPH (5‐hydroxy‐7‐(4 ^″^‐hydroxy‐3 ^″^‐methoxyphenyl)‐1‐phenyl‐3‐heptanone). These phytochemical compounds demonstrated potential anti‐inflammatory effects by suppressing the gene expression of key proinflammatory markers, including IL‐6, IL‐1*β*, and TNF‐*α*, in a concentration‐dependent manner as confirmed by q‐PCR. Molecular analysis further indicated that these substances, such as galangin and 5‐HHMPH, exhibit a strong binding affinity for the COX‐2 active site, highlighting their potential to modulate inflammation [32]. Interestingly, galangin exhibits significant anticancer potential against multiple cancers at different concentrations, particularly liver cancer (40–120 *μ*M) [33], breast cancer (0–100 *μ*g/mL) [34], lung cancer (20 *μ*M) [35], neuroblastoma (3.75 *μ*M) [36], gastric cancer (18.685 *μ*M) [37], and esophageal cancer (30 *μ*M) [37]. Despite its anticancer potential, its molecular mechanisms in different cancer types are not fully understood. Different studies suggest that galangin inhibits the viability of gastric cancer cells without harming normal gastric epithelial mucosal GES‐1 cells. It downregulated cell proliferation by reducing the protein expression of Ki67 and PCNA, and induced apoptosis by lowering BCL‐2 levels and upregulating cleaved Caspase‐3 and PARP levels as confirmed by western blotting. It also significantly inhibited the JAK2/STAT3 signaling pathway. Notably, overexpression of the STAT3 protein level reversed these effects, confirming that STAT3 suppression mediates the anticancer activity of galangin [37]. Another study showed that there was suppression in breast cancer cell proliferation, which was both time‐ and dose‐dependent, after treatment with the methanolic extract of *A. officinarum*. The extract hampers the advancement of cell cycle phases in breast cancer cells by downregulating key S‐phase regulatory proteins, including cyclin A, E2F1, and CDK2, as confirmed by flow cytometry. Moreover, the extracts were found to induce apoptosis mainly through mitochondrial‐dependent pathways and caspase activation, indicating their promising role in breast cancer treatment, confirmed by western blotting [38]. The rhizome extract of this plant has been suggested as a natural chemopreventive agent with strong potential against hepatocellular carcinoma (HCC) in rat models. When combined with cisplatin (CDDP), it functions as an effective chemosensitizer, enhancing the anticancer activity of the drug. Furthermore, the extract provides hepatic tissue integrity and improves hepatic function by decreasing the levels of alpha‐fetoprotein concentration in an experimental HCC model [39]. In liver cancer cells, glangal initiates apoptosis by promoting the translocation of Bax, a proapoptotic mitochondrial protein, leading to the release of cytochrome c and apoptosis‐inducing factor (IF) into the cytosol; these molecular changes were confirmed by western blotting [33]. In neuroblastoma, it activates Caspase‐3 by regulating the BCL‐2/BAX pathway, followed by apoptosis confirmed by mRNA expression studies through q‐PCR [36]. These studies confirm the effect of *A. officinarum* against multiple cancers and can be developed as a multifaceted cancer drug.

#### 3.2.3. *Antrodia cinnamomea*

*A. cinnamome*, a unique fungus native to Taiwan, has a rich history of applications in traditional medicine and as a health food supplement. This fungus has been valued for generations as a natural solution to various ailments [40, 41]. As a brown rot fungus with host specificity, it thrives within the heartwood of Cinnamomum kanehirai trees, rendering it rare in its natural habitat [40]. This rarity enhances its value for research and commercial applications [41]. It has traditionally been employed to treat conditions such as inflammation, diarrhea, liver disorders, drug poisoning, and cancer [42, 43]. This mushroom contains active metabolites such as polysaccharides, polyphenols, triterpenoids, and ubiquinone derivatives, which have applications in medicine and the food industry [40, 44, 45]. Recently, it has gained global attention because of its bioactive compounds and potential therapeutic applications [42, 45]. Mushroom metabolites, including polysaccharides, perform several roles, such as promoting growth, reducing inflammation, fighting cancer, protecting the liver and nerves, lowering blood pressure, and relaxing blood vessels [46]. *A. cinnamomea* polysaccharides can enhance the expression of heme oxygenase‐1, boost antioxidant activity, and counteract the inflammatory pathway controlled by nuclear factor kappa B (NF‐*κ*B). It also demonstrates notable therapeutic effects, particularly in protecting the liver and exerting anticancer effects [41]. The ethanol extract of AC has been shown to suppress breast cancer growth, including tamoxifen‐resistant forms, particularly when used in combination with chemotherapy. AC combined with tamoxifen was more effective in suppressing the growth of tamoxifen‐resistant breast cancer cells than *A. cinnamomea* alone [47]. This induces apoptosis and suppresses SKP2 mRNA expression by increasing miR‐30‐5p, miR‐26‐5p, and miR‐21‐5p levels in these cells, as confirmed by q‐PCR with the drug concentration of 185.043 *μ*g/mL [47]. Another study showed that *A. cinnamomea* extract suppressed the growth of lung cancer cells in a dose‐dependent manner. This induces cell cycle arrest in the S‐phase. The extract enhanced p53 expression and downregulated BCL‐2, consistent with mRNA expression. Cells treated with *A. cinnamomea* extract inhibited cancer cell migration and suppressed MMP‐9 and MMP‐2 expression at both the protein and mRNA levels, as confirmed by western blotting and q‐PCR, respectively. These findings underscore the potential of ACE as an adjuvant therapy for lung cancer [48].

#### 3.2.4. *Azadirachta indica*

*A. indica*, widely known as Neem, a member of the Meliaceae family, is a robust and rapidly growing evergreen tree native to subtropical and tropical regions and has been utilized as a medicinal plant for decades. This plant is indigenous to the Indo‐Pakistan subcontinent, particularly Southeast Asia, which includes India, Myanmar, Sri Lanka, Thailand, Malaysia, and Indonesia. Several parts of this plant have been exploited for various medicinal uses, such as leaves for ulcers, flowers for bile disorders, and bark for psychiatric disorders and the central nervous system (CNS). It is also recognized for its antiplasmodial properties and has been employed in treating smallpox and several other infectious diseases, in addition to being effective in pest control. Neem exhibits pharmacological activities, including insecticidal, anti‐inflammatory, antibacterial, and antioxidant properties [49]. The phytochemical constituents include terpenoids, flavonoids, coumarins, carbohydrates, proteins, and fatty acids [50]. Various studies have confirmed that neem components prevent the survival, angiogenesis, proliferation, metastasis, and invasion of cancer cells. The NF‐*κ*B signaling pathway in cancer cells was suppressed in nimbolide (a limonoid derived from) treated xenograft mouse models, leading to the downregulation of tumorigenic proteins as confirmed by western blotting. Polyphenols from neem also inhibit the expression of atherogenesis‐related genes in human mononuclear cells, as determined by q‐PCR [51]. The methanolic extract of Neem stem bark was found to induce cell cycle arrest and apoptosis in cervical cancer cells, as confirmed by flow cytometry at a drug concentration of about 115 *μ*g/mL. It also inhibits the migration of breast cancer cells, even at nontoxic dosages, suggesting its therapeutic potential, confirmed by the wound‐healing technique [52]. Similarly, methanolic neem stem bark extract (MNBE) specifically affects cervical cancer cells more than normal cells, causing transcriptional downregulation of cell cycle genes, such as cyclin A, cyclin B, CDK1a, and prosurvival genes, such as BCL‐2 and survivin, confirmed through q‐PCR. NFkB‐p65 was downregulated at the protein level, whereas active Caspase‐3 was upregulated, which was confirmed through western blotting [52]. Epoxyazadiradione (EAD), a significant limonoid purified from neem seeds, exhibits anticancer potential in human cervical adenocarcinoma cells. In addition, cervical adenocarcinoma cells treated with EAD showed DNA fragmentation, blebbing of the cell membrane, translocation of phosphatidylserine, Bax upregulation, increased caspase 3 activity, PARP cleavage, and BCL‐2 downregulation, indicating cell death via apoptosis, as confirmed by western blotting. Moreover, apoptosis in cervical cancer cells by neem extract was confirmed by the increase in Caspase 9 activity and the release of cytochrome C which was also confirmed by cytochrome kit assay as well as western blotting [53].

#### 3.2.5. *Basella alba*

*B. alba*, commonly referred to as Malabar spinach, is a rapidly growing, succulent leafy vegetable. It belongs to the family Basellaceae. This plant is indigenous to India, particularly in the southern region of Andhra Pradesh [54]. This plant is rich in minerals and vitamins and is of significant ethnomedical importance in traditional Indian medicine, exhibiting androgenic potential of antioxidant, antiviral, antibacterial, anti‐inflammatory, antiulcer, antidiabetic, and antidepressant properties. It also facilitates wound healing and exhibits nephroprotective and hepatoprotective activities. Several bioactive compounds isolated from this plant, such as hydroxymethyl colchicine, 10‐[methoxycarbonyl]‐N‐acetylcolchinol, and [22S]‐21‐Acetoy‐6a, 11 a‐dihydroxy‐16a, 17 a‐propylmethylenedioxypregna−1, 4‐diene‐3, 20‐dione, are known for their anti‐inflammatory properties. Additionally, flavonoids present in plants are known to exert anticancer effects. The methanolic extract of *B. alba* demonstrated antioxidant activity greater than that of ascorbic acid [55]. Fractions of *B. alba* plant extract, separated by gel filtration chromatography, have been shown to effectively induce apoptosis, as confirmed by immunofluorescence in Ehrlich ascites carcinoma (EAC) cells, proving their anticancer properties. Gene expression profiling of key apoptotic markers, such as BAX, BCL‐2, NF‐k*β*, PARP‐1, TP53, TNF‐*α*, Fas, Cyt‐C, and Caspase‐8, 9, and 3, through q‐PCR indicated that the plant triggers apoptosis through the activation of both extrinsic and intrinsic pathways [56].

#### 3.2.6. *Camellia sinensis*

*C. sinensis*, commonly referred to as tea, is a highly consumed drink worldwide. According to the literature, this plant originated in China, but it is currently consumed worldwide, with India, China, and Kenya being its primary producers. Phytochemical analysis has identified caffeine, amino acids, polyphenols, carbohydrates, minerals, and trace amounts of vitamins. The polyphenolic content is primarily influenced by the extent of fermentation, which alters both the type and concentration of these compounds [57]. The therapeutic effects of green tea are mainly due to polyphenolic compounds, such as epicatechin‐3‐gallate(ECG), epigallocatechin‐3‐gallate (EGCG), epigallocatechin (EG), and epicatechin. Among these, EGCG plays a key role, as it significantly reduces food intake, body weight, and serum levels of testosterone, insulin, insulin‐like growth Factor I, leptin, estradiol, LH, cholesterol, glucose, and triglyceride. Additionally, EGCG has been found to suppress the growth of hormone‐sensitive organs, such as the prostate, uterus, and ovary. The bioactive compounds in green tea are also effective in mitigating oxidative stress‐induced cellular damage, enhancing both cell‐mediated and humoral immunity, lowering the risk of certain cancers, and offering protective advantages in inflammatory disorders [58]. The chemopreventive effects of green tea are mainly mediated by EGCG, which promotes apoptosis and cell cycle arrest by modulating cell cycle proteins, activating caspases, and inhibiting NF‐*κ*B signaling, as confirmed by different assays such as flow cytometry, immunohistochemistry, and transfection luciferase reporter assay. EGCG also upregulates IL‐23‐mediated DNA repair and stimulates T‐cell cytotoxic activity in the tumor microenvironment, as observed through ELISA. Moreover, it suppresses cancer development by targeting different key signaling mechanisms involved in inflammation, cell proliferation, transformation, and metastasis [59]. Polyphenols, especially catechin and EGCG, have been shown to influence angiogenesis and tumor growth signaling biomarkers in TNBC. The extract specifically reduced the levels of VEGF, as observed through ELISA, a key mediator of tumor angiogenesis, and hepatocyte growth factor (HGF), which plays a key role in tumor growth, migration, and invasion at a dose concentration of 5–10 *μ*M [60]. Catechin‐rich green tea leaves promote apoptosis and suppress angiogenesis in colon cancer cells by lowering VEGF expression and inhibiting its promoting activity, as confirmed by transfection with luciferase reporter assay [61, 62]. The leaves also exhibit inhibitory effects on MMP‐9 in colon cancer cells, confirmed by immunohistochemistry assay, contributing to reduced invasiveness and metastatic potential at drug concentrations of 0–5 *μ*M [62]. Moreover, EGCG suppresses the growth and promotes the regression of prostate (600 *μ*M) and breast tumors (concentration of 23.4 *μ*g/mL) by influencing hormonal regulation within the endocrine system [60].

#### 3.2.7. *Erycibe elliptilimba*

*E. elliptilimba*, as described by Merr. and Chun, a medicinal plant indigenous to Thailand and classified under the Convolvulaceae family, is traditionally utilized in Thai medicine to mitigate fever symptoms associated with infections and inflammation. The genus *Erycibe* of the Convolvulaceae family has demonstrated various therapeutic effects, such as anti‐inflammatory, analgesic, anticancer, hepatoprotective, and antioxidant properties. A fraction of the methanolic extract of *E. elliptilimba* demonstrated cytotoxic and antiproliferative effects on Her2+ breast and melanoma cancer cells [63]. Cell cycle analysis revealed that the growth of the exposed cells occurred with a concentration of 56.07 ug/mL, as validated by the trypan blue exclusion assay. The findings suggest that this plant exerts an antiproliferative effect on melanoma by inducing arrest at the G2/M phase of the cell cycle, indicating a potential interaction with tubulin, similar to that of other plant‐derived chemotherapeutic agents. However, the antiproliferative activity may vary depending on the cell type and culture conditions [63].

#### 3.2.8. *Eryngium billardieri*

*E. billardieri*, is an herbaceous plant classified within the Apiaceae family and is the most prevalent and widely distributed species in Iran. Its range extends across Caucasia, Northeastern Anatolia, Northern and Central Asia, Southern Russia, Western Himalayas, Eastern and Central Iran, Afghanistan, and Pakistan. The key elements present in *E. billardieri* are saponins, alkaloids, terpenoids, flavonoids, caffeic acid, and *β*‐carotene. These substances have been shown to exhibit a variety of therapeutic effects, including antibacterial, anti‐inflammatory, cytotoxic, antifungal, antiapoptotic, and antimalarial properties [64]. Different parts of this plant, such as the roots and leaves, are used in Iran to treat various conditions, including wound healing, urinary tract infections, rheumatism, and inflammatory disorders [65]. Furthermore, *E. billardieri* has been proposed as a potential therapy for pancreatic cancer because of its cytotoxic and apoptotic properties. Research on the effects of *E. billardieri* has shown that n‐hexane, DCM, and methanol extracts with a dose concentration of 50–200 *μ*g/mL have promising effects on pancreatic cells by regulating apoptosis. The DCM and n‐hexane extracts of this plant exert high expression of apoptosis; however, the number of necrotic cells after this treatment was very low, which indicates programmed cancer cell death in response to the extracts of *E. billardieri.* Apoptosis is regulated by increasing the expression of Bax mRNA, confirmed by the q‐PCR technique [66]. Cyclin D1 also significantly contributes to the progression of specific human cancers [67, 68]. Extracts from this plant have been shown to reduce cyclin D1 levels in cancer cells, thereby inhibiting their growth, with no impact on normal cells [66].

#### 3.2.9. *Grammatophyllum speciosum*

*G. speciosum,* is a large orchid species in the Orchidaceae family, predominantly found in the tropical forests of Southeast Asia, including Malaysia, the Philippines, Indonesia, Burma, Thailand, and Laos. In traditional Thai medicine, an infusion of *G. speciosum* is used to alleviate ailments such as sore throats and bronchitis. The pseudobulb of this orchid, when immersed in ethanol, is revered by locals as an “elixir of everlasting life.” Historically, it has been used to treat skin rashes and abscesses in traditional medicine. Phytochemical investigations of pseudobulbs have identified several compounds, including gastrodin, Vandateroside II, grammatophyllosides A‐D, orcinol, cronupapine, (R)‐2‐benzylmalic acid, vanilloloside, and (R)‐eucomic acid. It comprises glucosyloxybenzyl derivatives. These phytochemicals have demonstrated various medicinal properties, such as antioxidant, anticonvulsant, antiapoptotic effects, memory enhancement, and protective benefits against osteoporosis [69]. The antiproliferative impacts of the ethanolic extract from *Grammatophyllum speciosum* pseudobulb (GSE) and isovitexin (a bioactive component) were observed in the ER+ breast cancer cells at the dose concentration, which ranged from 100 to 1000 *μ*g/mL. *G. speciosum* has been observed to reduce AKT/*β*‐catenin pathway activity observed through western blotting indicating its potential antiproliferative effects [70]. The study indicated that nontoxic concentrations of GSE markedly suppressed the growth of breast cancer cells, accompanied by a decrease in both the number and size of tumor colonies. GSE therapy inhibited the levels of phosphorylated *β*‐catenin and AKT in these cells. Additionally, isovitexin‐treated breast cancer cells also exhibited antiproliferative effects, as evidenced by the inhibition of colony formation and decreased levels of phosphorylated AKT and *β*‐catenin expression in proteins observed through the western blotting [70]. The tumorostatic and tumorotoxic effects of *G. speciosum* extracts can be developed as a therapy for breast cancer in combination with conventional treatments [70].

#### 3.2.10. *Justicia adhatoda*

*J. adhatoda*, commonly referred to as Malabar nut, is a tropical shrub from the Acanthaceae family. It is native to various regions of Southeast Asia, including Afghanistan, Pakistan, India, Nepal, Bangladesh, Vietnam, Myanmar, Laos, and the Eastern Himalayas. For over two millennia, it has been an integral part of Indian medicine. In traditional Ayurvedic practice, it is widely used for treating coughs, colds, asthma, tuberculosis, and other pulmonary diseases [71]. Phytochemical investigations have revealed that the plant contains pyrro‐quinazoline alkaloids, including vasicine, vasicol, vasicinone, peganine, and betaine, as well as steroids, carbohydrates, and alkanes [72]. The phytochemicals present in this plant exhibit diverse therapeutic activities, including antibacterial, antiprotozoal, anti‐inflammatory, abortifacient, antiasthamatic, and hypolipidemic activities. The plant extract exhibits antitumor properties against breast cancer cells by inducing the activation of apoptotic markers, such as Bax, cleaved PARP, and Caspase‐3, as analyzed by western blotting, thus promoting apoptosis at a drug concentration of 161.57 *μ*g/mL [73]. The methanolic extract of *J. adhatoda* significantly elevated NO and ROS production in breast cancer cells, determined by the NO detection kit and immunofluorescence assay, respectively. Cell cycle analysis by flow cytometry showed arrest at the sub‐G0 phase, indicating apoptotic cell death. The extract also disrupts the mitochondrial membrane potential and effectively inhibits cell migration and colony formation, demonstrating its potential to impair cancer cell survival and metastatic behavior [73]. Compared with paclitaxel (PTX), a conventional drug used in breast cancer therapy, the extract showed similar therapeutic efficacy [73]. This plant has been shown to induce autophagy in human colorectal cancer cells, as marked by the conversion of LC3‐1 to LC3‐II, confirmed by the western blotting and immunofluorescence at the drug concentration of 1 *μ*M. Formation of LC3 puncta, autophagic vesicles, and downregulation of SQSTM1/p62. In xenograft models, oral administration of *J. adhatoda* extract to NOD‐SCID mice with colorectal tumors led to reduced levels of p‐PDK1, p‐mTOR, and p‐p70S6k/p‐RPS6KB2, while upregulating class III PI3K, Beclin 1, Atg5‐Atg12, and mitochondrial BNIP3, indicating enhanced autophagy signaling. Additionally, pretreatment with 3‐methyladenine or Atg5 shRNA attenuated *J. adhatoda* induced LC3‐II expression and puncta formation, confirming the role of class III PI3K and Atg5 in mediating its autophagic effects. These effects were confirmed through the Western blotting technique [74].

#### 3.2.11. *Lagerstroemia speciosa*

*L. speciosa*, is a deciduous tropical plant commonly known as the Queen′s Crape Myrtle, Pride of India, or Banaba. This plant is a member of the Lythraceae family and is native to Southeast Asia. It is distinguished by the presence of purple to pink flowers and smooth, mottled bark. Historically, *L. speciosa* has been used for both ornamental and medicinal purposes [75]. Various parts of this plant are used to treat diabetes, diarrhea, and urinary tract infections in traditional medicine [76]. The plant has attracted scientific attention for its antidiabetic properties, primarily due to corosolic acid, a compound studied for its role in regulating blood glucose levels [77]. The pharmacological activities associated with this plant have been widely studied, particularly its antioxidant, antimicrobial, and anti‐inflammatory activities. Among its key constituents, corosolic acid (2*α*‐hydroxyursolic acid), a pentacyclic triterpenoid, has demonstrated diverse therapeutic attributes, including antidiabetic, antiobesity, antihyperlanticancer, antiviral, and anticancer effects. Structurally related compounds, such as ursolic acid (UA), maslinic acid (MA), asiatic acid (AA), oleanolic acid (OA), and betulinic acid (BA), are also natural pentacyclic triterpenes derived from various plants and share similar profiles [78]. In various cancer types, such as breast cancer [79], pancreatic [80], hepatic [81], colon [82], lung [83], renal, and prostate [84] cancers, corosolic acid demonstrates anticancer effects by modulating the apoptotic pathway via different signaling cascades [78]. This phytochemical and its analogs also regulate the angiogenic pathway in certain cancer types, such as lung, liver, and kidney cancers, to impart antitumor effects. Corosolic acid suppressed the proliferation of ER+ and TNBC cells (dose concentration from 5 to 50 *μ*M) but selectively induced apoptosis only in TNBC cells. It activates caspase‐dependent apoptotic pathways in TNBC, confirmed with the q‐PCR and western blotting, whereas no significant changes in apoptotic markers are observed in ER+ cells [85]. Apoptosis in TNBC cells was induced by the suppression of phosphorylated JAK2, and STAT3 was analyzed through western blotting, indicating inhibition of the JAK2/STAT3 signaling pathway. CRA induced apoptosis by increasing the levels of procaspase‐8, 9, and 3, along with PARP cleavage. This caspase‐dependent cell death was confirmed by the inhibitory effect of z‐VAD‐FMK, a pan‐caspase inhibitor. Furthermore, CRA treatment upregulated proapoptotic proteins (Bax, Fas, and FasL) and reduced the levels of antiapoptotic proteins (BCL‐2 and survivin), highlighting its role in modulating the apoptotic pathway [86]. These expression studies were confirmed by the western blotting technique.

#### 3.2.12. *Millettia pinnata*

*M. pinnata* tree, also known as pongamia pinnatae and commonly known as Indian Beech or Karanj, is a tree from the Fabaceae family, widely distributed across Indian forests. It holds significant ethnomedicinal value and is extensively used by tribal people for its therapeutic benefits. In Ayurvedic medicine, the entire plant is traditionally used to manage various diseases. Additionally, crude extracts have been reported in scientific studies for their therapeutic potential against conditions such as tumors, tooth decay, piles, ulcers, scabies, gonorrhea, rheumatism, leukoderma, and vaginal and skin diseases [87]. *M. pinnata* leaf extract contains phytoconstituents that may have antibacterial, antioxidant, and anticancer properties. The extraction and characterization of the chemical composition of *M. pinnata* leaf extract, as well as the molecular targets in cancer cells, need to be examined [88]. Pongamal, a flavone derivative from *M. pinnata* seed extract, exhibits anticancer properties [89]. The methanolic extract of *M. pinnata*, particularly pongapin, exhibits inhibitory activity against CYP1A1, confirmed by luminescence‐based assay at a dose concentration of 4.4 *μ*M. In breast cancer with overexpressed CYP1A1, the methanolic extract can induce the cell cycle at the G0–G1 phase, as revealed by flow cytometry, and significantly reduce cyclin D1 protein expression as observed by the western blotting technique, indicating its potential as an antiproliferative agent [90].

#### 3.2.13. *Panax notoginseng*

*P. notoginseng*, also known as Sanqi or Sanchi in Chinese, is a medicinal herb from the Araliaceae family, traditionally used to improve blood circulation. Initially utilized by minority ethnic groups in Southwest China, its use became widespread during the Ming Dynasty and is now common throughout Asia. This herb is cultivated in the Chinese provinces of Yunnan, Guangxi, Jiangxi, and Sichuan [91]. Phytochemical studies have identified several beneficial compounds, including saponins, ginsenosides, aminoglycosides, and volatile oils, which exhibit anti‐inflammatory, anticancer, antifungal, antidiabetic, and neuroprotective properties [92]. Ginsenosides Rg3 and Rh2 have demonstrated the ability to inhibit tumor cell proliferation, promote cell differentiation and apoptosis, and suppress tumor invasion and metastasis [93]. Immunoblotting analyses indicated that R7 suppresses AKT phosphorylation at Ser473 and Thr308, with dose concentrations of 10.27 *μ*g/mL indicating its potential as a chemotherapeutic agent for cervical cancer and other tumors associated with the PI3K/PTEN/AKT/mTOR signaling pathway [94]. It was also observed that *P. notoginseng* derivative, notoginsenoside R7, induced apoptosis and suppressed cervical adenocarcinoma cell proliferation in an in vitro system. In silico docking studies suggest that R7 may directly bind to AKT [94]. PNS significantly inhibited the proliferation and apoptosis of retinoblastoma cells. Treatment resulted in upregulated mRNA and protein expression of Caspase‐3, 8, 9, and Bax, along with increased levels of cleaved caspases, observed by both q‐PCR and western blotting techniques. Although total PI3K and AKT1 levels remained unchanged, p‐PI3K, p‐AKT, and m‐TOR levels were significantly reduced (*p* < 0.05), and PTEN expression was notably increased (*p* < 0.01). Furthermore, the effects of PNS were consistent with those observed following PI3/AKT pathway inhibition using LY294002, suggesting that PNS mediates its anticancer activity by suppressing the PI3/AKT/mTOR pathway [95]. Consistent with the in vitro findings, R7 activated the proapoptotic BCL‐2 family and caspase family members in a mouse xenograft model, primarily by targeting PI3K (PTEN) and AKT, as observed through the western blotting technique. R7 treatment also led to reduced expression of raptor (regulatory‐associated protein of mTOR) and decreased mTOR protein phosphorylation, whereas total mTOR levels remained the same, indicating pathway‐specific inhibition through PTEN activation [94].

#### 3.2.14. *Pseuderanthemum palatiferum*

*P. palatiferum* (Nees), a member of the Acanthaceae family, was first identified in northern Vietnam in 1990. In traditional Vietnamese and Thai medicine, its leaves have been used to treat hypertension, diabetes, colitis, cancer, nephritis, and anti‐inflammatory diseases [96]. The plant contains various bioactive compounds, including apigenin, stigmasterol, *β*‐sitosterol, triterpenoids, kaempferol, phytol, saponin, and salicylic acid, along with essential amino acids such as threonine, methionine, and lysine [97]. The ethanolic extract of PP with the dose concentration of 100–500 *μ*g/mL disrupted the mitochondrial transmembrane potential of TNBC cells, suggesting the activation of the mitochondrial‐dependent cell death pathway, involving both apoptosis and necrosis, determined by flow cytometry [98]. It also elevated cytosolic Ca2+ levels, indicating Ca2+ overload and involvement of endoplasmic reticulum (ER) stress, accompanied by the activation of Caspase‐3, 8, and 9, as measured using a spectrophotometer. By triggering both intrinsic and extrinsic signaling pathways, the fresh leaf ethanolic extract caused TNBC cells to undergo oxidative stress and ER‐induced programmed cell death [98].

#### 3.2.15. *Tylophora tanakae*

*T. tanakae*, which is a part of the Asclepiadaceae family, is indigenous to Japan′s Ryukyu Islands. *Tylophora* has long been utilized in local and ancestral medicine to treat a range of conditions, including bronchial asthma, bronchitis, cough, indigestion, wounds, ulcers, liver disorders, and as an expectorant [99]. This plant contains phenanthroindolizidine alkaloids, primarily isotlylocrebrine and tylophorine, which exhibit cytotoxic properties. Plant extracts can inhibit protein biosynthesis in cervical cancer cells [100]. Alkaloids derived from *T. tanakae* have demonstrated cytotoxic effects against both drug‐sensitive and drug‐resistant human cervical cancer cells at 7 nM drug concentration. Research findings indicate that these alkaloids exhibit significant cytotoxicity comparable with that of contemporary cytostatic drugs [101]. This plant extract also exhibits antiproliferative activity against adult T‐cell leukemia at a drug concentration of 0.5–100 *μ*g/mL [102].

### 3.3. Traditional Plants of European Origin

The research conducted on plants of European origin mostly shows anticancer activity affecting the increase in cancer cells by targeting the apoptotic pathway (Figure 2). In the following section, we discuss the plants whose molecular pathways have been studied for their anticancer activity (Figures 2 and 3).

#### 3.3.1. *Astragalus hamosus*

*A. hamosus*, is an annual or biennial herbaceous plant belonging to the family Angiosperms. It is native to central and southwest Asia, Southern Europe, the Mediterranean region, and the Caucasus. Traditionally, it has been used in herbal medicine for its pain‐relieving and anti‐inflammatory effects, as an emollient, demulcent, aphrodisiac, diuretic, and laxative. It is beneficial for conditions such as inflammation, ulcers, leukoderma, mucous membrane irritation, nervous disorders, and cataracts. Phytochemical investigations have identified flavonoids, phenols, and essential oils in this plant species. It exhibits anti‐inflammatory, antiproliferative, and cytotoxic effects. A combination of the two saponin compounds from this plant demonstrated antineoplastic activity against ER+ and triple‐negative breast cancer (TNBC) cells. Certain saponins extracted from *A. hamosus* have demonstrated a dose‐dependent ability to influence lymphocyte proliferation with dose concentration in human breast (253.2 *μ*g/mL), prostate (40.78 *μ*g/mL), and lung (1–50 *μ*M) cancer cell lines [103–105]. Investigation of the growth‐inhibitory effect of this plant on ER+ breast cancer cells indicated significant damage to the cells, leading to apoptosis. The extract also induced cell cycle arrest, as well as was analyzed by flow cytometry. The treatment initiated the decline of Ki67 and BCL‐2 and upregulation of Caspase‐9, 8, and 3 genes quantified by q‐PCR in ER+ breast cancer cells. Therefore, *A. hamosus* extract may serve as a therapeutic agent for breast cancer owing to its antiproliferative effects [106].

#### 3.3.2. *Vaccinium myrtillus*

*V. myrtillus*, commonly known as bilberry, is a perennial shrub native to central and northern Europe and belongs to the family Ericaceae. It typically grows close to the ground level. This plant is also found in some regions of Asia and North America [107]. Phytochemical analysis indicated the presence of bioactive compounds, mainly phenolic compounds, terpenoids, flavonols, tannins, and other molecules. The majority of anthocyanins include delphinidin and cyanidin [108]. The plant has various biological benefits, including anticancer properties. Studies using ER^+^ breast cancer cells have shown the impact of bilberry extract on reducing cellular proliferation in connection with its capacity to trigger cell rounding, apoptosis, and affect the organization and assembly of microtubules, leading to the formation of distinct punctate tubulin aggregates inside the cells [109]. Bilberry extract causes the accumulation of cells at mitosis and the G2/M stage as analyzed by flow cytometry, by a direct action on microtubules that only occurs at elevated extract levels (0.5 mg/mL and higher). Ethanolic extracts from bilberries also showed a suppressive effect on benzo(a)pyrene‐induced mutated breast cancer cells in vitro. It showed an inhibitory effect on the genes involved in migration and cell motility by inhibiting the NF‐*κ*B and PI3/AKT signaling pathways in TNBC cases, as observed through western blotting with a dose concentration of 30 *μ*g/mL [110, 111]. In vivo studies have shown that the intake of 5% concentrated blueberry powder inhibits cell proliferation by suppressing the Wnt/*β* signaling pathway [111].

#### 3.3.3. *Rosmarinus officinalis*

*R. officinalis* is a medicinal plant classified within the Lamiaceae family that originates from the mild climates of Mediterranean areas, such as Portugal [112]. Rosemary (*R. officinalis*) is a therapeutic herb commonly used in culinary applications [113]. Recent studies have demonstrated the pharmacological potential of curcumin in cancer chemoprevention and therapy. It contains three primary active compounds, carnosic acid (CNA), carnosol (CS), and rosmarinic acid (RA), which can enhance the antiproliferative effects of CDDP against cancer. Studies have assessed the antiproliferation properties of rosemary extract (RE), demonstrating an increased antiproliferation effect against human ovarian cancer cells, as well as on their CDDP‐resistant derivatives. Ovarian cancer cells consistently exhibited greater sensitivity to CS, CNA, and RA than resistant daughter cells. Both CS and RA exhibited enhanced antiproliferative effects when combined with CDDP against ovarian cancer cells. However, after RE underwent ultrafiltration, dialysis, and phenolic compound removal, RE lost its antiproliferative properties, indicating that these effects are primarily attributed to the phenolic compounds present in the plant extract [114]. Research on RE has demonstrated its ability to suppress ovarian cancer cell spread by disrupting various stages of the cell cycle. The extract promoted apoptosis by regulating multiple apoptosis‐related genes, highlighting its potential as a valuable complementary agent in cancer chemotherapy [114]. We found that BCL‐2, an antiapoptotic protein, was downregulated upon RE treatment at 2.5–20 *μ*g/mL. Cytochrome c, which is released from the mitochondria in response to proapoptotic stimuli, was found to be upregulated, leading to overall apoptosis of the cells, obtained through the apoptosis kit method [114].

### 3.4. Traditional Plants of African Origin

A few plants of African origin have also been studied recently to explore the mechanisms underlying their anticancer effects. The plants are discussed in the following section.

#### 3.4.1. *Terminalia brownii*

*T. brownii*, a part of the Combretaceae family, is a medicinal tree indigenous to several East African countries, including Ethiopia, Kenya, the Democratic Republic of Congo, Eritrea, Tanzania, Somalia, Sudan, and Uganda. Phytochemicals derived from the stem bark of *T. brownii* include triterpenoids, terminalianone, gallotannins, ellagitannins, punicalagin, terchebulin, and methyl‐(S)‐flavogallonate [115]. These phytochemicals are used to treat various health issues, such as yellow fever, diabetes, kidney problems, diarrhea, rheumatic conditions, stomach ulcers, allergic reactions, wounds, backaches, abdominal pain, malaria, and bronchial coughs [116]. Substances found within *T. brownii can* stop the growth of cancer cells [117]. Plant extracts were more effective at inhibiting ER^+^ breast cancer cell viability at high dosages ranging from 20–25 *μ*g/mL. Additionally, the common fruit extract malvidin reduced the migration of colon carcinoma cells with a dose concentration ranging from 15 to 20 *μ*g/mL. Furthermore, the chloroform, ethyl acetate, and hexane extracts demonstrated notable alterations in the ability of ER^+^ breast cancer, HCC (dose concentration ranging from 11.6 to 15 *μ*g/mL), and colon cancer cells, respectively, as observed in cell cycle analysis through flow cytometry to replicate through the S phase of the cell cycle [117]. [118], revealed that green silver nanoparticles isolated from the aqueous outer bark of *T. brownii* exhibited cytotoxic effects on TNBC cells with a drug concentration of about 115 *μ*g/mL. The TB‐AG nanoparticles induce cytotoxicity by decreasing membrane permeability and upregulating ROS, which can damage DNA and ultimately result in cell death by activating the Caspase‐3 apoptotic pathway, as confirmed by western blotting assay [118].

#### 3.4.2. *Triumfetta welwitschii*

*T. welwitschii*, is a long‐living herbaceous species of the Tiliaceae family that is commonly used in ethnomedicine [119]. This plant is native to various regions in Africa. In South Africa, an herbal decoction made from it is used to treat fevers, indicating its potential antipyretic effects [120]. *T. welwitschii* is a plant traditionally used to manage conditions such as fever and diarrhea, and has antibacterial activity. Recent studies on *T. welwitschii* extract revealed its anticancer activity against acute T cell leukemia, and it was found that the effects could not be reversed. *T. welwitschii* reduced cell viability at a dose concentration of 19 *μ*g/mL, with the effect increasing with both the concentration and duration of exposure. The extract induced apoptosis in acute T cell leukemia, as evidenced by DNA fragmentation, as observed in the gel migration assay. Additionally, when *T. welwitschii* was combined with reduced glutathione (GSH), there was a significant decrease in the growth of acute T cell leukemia cells compared with that in untreated cells. This finding was surprising because cancer cells typically exhibit higher GSH levels than normal cells. The study′s outcomes indicate that *T. welwitschii* could be a promising source of constituents with potential as anticancer agents [121].

### 3.5. Traditional Plants of American Origin

Very little research has been conducted on the traditional plants of the American subcontinent. In the following section, we discuss only those plants whose mode of action in various cancers has been studied by different research groups.

#### 3.5.1. *Turnera diffusa*

*T. diffusa*, commonly referred to as Damiana, is a diminutive shrub of the Turneraceae family [122]. It flourishes in the tropical and subtropical areas of America. The ancient Maya used it to address dizziness and balance disorders. Mexican Indigenous people have historically prepared a drink from its leaves, believing in its supposed aphrodisiac properties [123]. Phytochemical analysis identified the presence of bioactive compounds, including phenolics such as phenolic acid derivatives, cyanogenic glycosides, flavonoids, various fatty acids, essential oils, alkaloids, and s*μ*gar‐linked molecules, primarily sourced from leaves and stems [124]. *T. diffusa* has hypoglycemic, antiaromatase, prosexual, estrogenic, antibacterial, and antioxidant properties that have been demonstrated in pharmacological studies. The methanol‐based extract from *T. diffusa* showed activity against various cancer cell lines in four of its organic parts. Two active chemicals, apigenin and arbutin, were extracted from the most active fractions. Studies have assessed the potential cytotoxic impact of the extracts and organic fractions of this plant on epithelial tumor cell‐like with different dose concentrations (cervical squamous carcinoma, cervical carcinoma (50.14 *μ*g/mL), and HCC (43.87 *μ*g/mL), TNBC (30.67 *μ*g/mL), epithelial breast cancer (54.02 *μ*g/mL). The apigenin activity of *the methanolic extract of T. diffusa* is partially responsible for its cytotoxic action on TNBC [125]. Arbutin is another phenolic compound in the methanol‐derived extract of *T. diffusa* that exhibits toxic effects targeting cancer cells. In ER+ breast cancer cells, arbutin induced apoptosis via the p53 and Caspase 3 pathway. It also activates the estrogen receptor and alpha signal transduction pathways and promotes the upregulation of inflammation‐promoting cytokines. Additionally, arbutin exhibited cytotoxicity in cervical cancer cells. Higher doses of this compound can increase oxidative stress, inflammation, and genotoxicity [126].

#### 3.5.2. *Annona muricata*

*A. muricata*, commonly termed as soursop, belongs to the Annonaceae family. Indigenous to the warm tropical regions of South and North America, it is now extensively harvested globally in regions with tropical and subtropical climates. *A. muricata* is a year‐round leafy tree that reaches a height of 5–8 m and is distinguished by its open, rounded crown and large, shiny, dark green foliage. The fruit is edible and is a collective ovoid berry with a dark green exterior. Different sections of the plant have traditionally been utilized for healing and therapeutic applications, with decoctions from the bark, roots, seeds, and leaves being the most prevalent. In sub‐Saharan regions, *A. muricata* is used to manage health conditions, including malaria, stomach aches, parasitic infections, diabetes, and cancer. The fruit is consumed for relief from arthritis, nerve pain, digestive disturbances, rheumatism, dysentery, fever, parasitic infections, skin rashes, and intestinal worms, and is believed to enhance breast milk production. Leaves are used to address conditions such as cystitis, diabetes, headaches, and insomnia, whereas crushed seeds are considered to possess anthelmintic properties. Phytochemical analysis of this plant extract identified 212 bioactive compounds, including acetogenins, alkaloids, phenols, essential oils, carotenoids, amides, and flavonoid cyclopeptides as some of the functional compounds. The seeds and leaves contain enzymatic antioxidants, vitamins C and E, cyclopeptides, and N‐p‐coumaroyl tyramine, demonstrating both anti‐inflammatory and antitumor effects. The mechanism underlying the cytotoxicity of the extract may involve mitochondrial membrane disruption, cell cycle arrest, apoptosis, ROS generation, and a decrease in the expression of the antiapoptotic marker BCL‐2, as confirmed by the q‐PCR technique [127]. The water extracts reduced prostate cell proliferation and promoted apoptosis by elevating Bax levels and reducing BCL‐2 levels, as observed by immunohistochemistry. It has been shown to have protective effects against DNA damage in DMBA‐induced breast tissue cell proliferation in mice. *Annona muricata* ethyl acetate (AMEA) significantly decreased cell viability and NF‐*κ*B and p65 protein expression in breast cancer cells with a dose concentration of 1–100 *μ*g/mL, as identified by the MTT assay. Immunoblotting findings indicate that AMEA exerts antiproliferative effects by influencing EGFR‐dependent pathways, such as the suppression of AKT, MAPK, NF‐*κ*B, and cyclin D1 activity, measured by western blotting [128]. AMEA extract also induced G1 phase cell cycle arrest and exhibited significant cytotoxic effects on colon cancer cells, as determined by flow cytometry assays with a dose concentration of 8.98–11.43 *μ*g/mL. EEAM treatment induced apoptosis via BCL‐2 downregulation, excessive ROS accumulation, MMP disruption, cytochrome c leakage, and caspase activation in both colorectal adenocarcinoma and colorectal carcinoma cells, as observed by immunofluorescence. EEAM also suppresses the movement and infiltration of colorectal adenocarcinoma and colorectal carcinoma cells [129]. Metabolic profiling of the graviola fruit pulp extract combined with ionic liquid (IL‐GPE) revealed that IL‐GPE alters several metabolic pathways, including amino acid metabolism, oxygen‐dependent glycolysis, the urea cycle, and ketone body metabolism in colon cancer cells (HT29). These pathways are involved in energy metabolism and cancer cell growth [130]. Studies have also shown that the anticancer activity depends on the location of the medicinal plant. The MTT assay of 19 samples of *A. muricata* from various locations on different subtypes of breast cancer, such as ER^+^ and TNBC, showed that the IC50s of the samples differed [131]. The chloroform extract of *A. muricata* (AMCE) caused TNBC cells to undergo apoptosis in both in vitro and in vivo studies, decreased tumor size and mass, and exhibited antimetastatic properties. Additionally, it increased the population of immune cells, including T lymphocytes and natural killer cells, while decreasing the levels of malondialdehyde and nitric oxide in the tumor. As the different extracts of AM show cytotoxic effects on different cancers, such as colon and breast cancer, it has a strong profile for use as a cancer treatment [131].

#### 3.5.3. *Lantana camara*

*L. camara*, a prominent weed, is a low, erect, rugged, hairy, evergreen shrub of the Verbenaceae family, native to the tropical regions of America [132]. It has been used in traditional and indigenous medicine for centuries to treat various ailments, such as asthma, whooping cough, bronchitis, arterial hypertension, colds, headaches, uterine hemorrhage, chicken pox, eye injuries, and coughing [132]. Ethnomedical and scientific literature highlights the medicinal properties of *L. camara*, underscoring its value. Pentacyclic triterpenoids, oleanolic and UAs, are among the numerous active compounds in this important medicinal herb [133]. LC‐MS/MS analysis of the methanol‐based extract of the plant identified four compounds: icterogenin, lantadene A, B, and C. Studies have demonstrated that the metabolites identified in plants exhibit biological activity against breast cancer, including cytotoxicity, antiproliferation, and apoptosis [134]. *L. camara* demonstrated cell death properties like morphological changes, DNA fragmentation, oxidative stress, and increased caspase activity in the ER^+^ and PR+ human breast cancer cells [135]. Observations reveal that the apoptosis triggered by the *L. camara* extract treatment was controlled by suppressing NF‐*κ*B activity, which led to lowering the key downstream protein, the BCL‐2 family, with an increase in Bid and Bax, as observed by western blotting [135]. By suppressing NF‐*κ*B activity, the extracts also led to a reduction in cyclin D1 and VEGF levels, thereby inhibiting cell proliferation and angiogenesis [136]. *L. camara* extract regulated the cleavage of Caspase‐9, Caspase‐8, and PARP in ER+ breast cancer cells, observed by western blotting at a dose concentration of 46.63 *μ*g/mL [135]. The leaves of this plant also inhibit the migratory ability of TNBC cells at a dose concentration of 40–150 *μ*g/mL [137]. Research studies indicate that *L. camara* extract holds promise as an agent for breast cancer [135].

#### 3.5.4. *Gossypium hirsutum*

*G. hirsutum*, also known as upland cotton, is the most extensively cultivated variety, accounting for 90% of global cotton production. It is native to the coastal region of Yucatán, Mexico, and is more sparsely found in nearby areas, extending as far north as the Florida Keys. This plant was cultivated approximately 5000 years ago [138]. Gossypol and its derivatives are the primary lipid‐soluble polyphenols with anticancer properties in colon cancer [139]. The mRNA levels of many indicators implicated in glucose transport, lipid biosynthesis, inflammatory response, and cancer development, which are examined in human colon cancer cells treated with bacterial endotoxin lipopolysaccharides (LPS), bioactive extracts, and gossypol produced from cottonseed, displayed a significant change in their expression levels [140]. In breast cancer cells, low doses of gossypol, as low as 30 nM, exhibit potent antiproliferative activity [141]. Ye et al. demonstrated dose‐dependent inhibition of breast cancer cell growth when gossypol‐enriched cottonseed oil was administered. The signaling pathway involved in this action is through reducing the BCL‐2 gene expression both at the transcriptional and translation levels, observed by q‐PCR and western blotting technique, ultimately triggering apoptotic cell death [142].

## 4. Safety and Translational Considerations

For the effective development of the medicinal plants with anticancer potential for clinical use requires careful evaluation of their safety, toxicity and consistency. Although many plant extracts show cytotoxic activity in vitro, these effects may not translate into effective in vivo outcomes. Therefore, standardizing preparations and through toxicological assessment is essential. Additionally, chemo‐typic and geographical variations can alter the phytochemical composition of the plants, influencing their efficacy and toxicity. This variability highlights the need for marker compounds and rigorous quality control procedures [143]. Standardizing extracts with respect to plant part, extraction solvent, and key constituents is important to achieving consistent biological effects [144]. Organ‐specific toxicities must also be considered, since several plant‐derived compounds display narrow therapeutic windows, with toxic effects emerging at higher doses or with prolonged use [145–148]. Systematic toxicological studies and early‐phase clinical trials are required to define safety margins, identify contraindications, and inform rational dosing. Potential interactions with conventional chemotherapy are of particular concern, as phytochemicals may modulate drug metabolism and therapeutic efficacy [149]. Although certain combinations might confer synergistic benefits, clinical evidence remains limited [150]. The current abundance of in vitro data risks overinterpretation, as the concentrations effective in cell‐based assays may not be achievable or safe in humans [151]. Until robust clinical data are available, these plants should be regarded as experimental leads rather than established oncologic therapies [151]. These safety considerations are crucial for traditional medicinal plants translating into evidence‐based oncology.

## 5. Application in Therapeutics

Cancer is one of the deadliest illnesses that still affects a substantial portion of the global population. Numerous plant‐derived compounds, including, camptothecin (CPT), PTX, vincristine, and vinblastine, have become the basic components of contemporary cancer therapies, as extensively documented in scientific research. These natural compounds are generally regarded as safe and well tolerated at therapeutic levels compared with their synthetic alternatives, leading to a substantial increase in their demand. Despite the growing interest in powerful phytoconstituents, formulation developers encounter several difficulties during the drug development process, including marginal permeability, low bioavailability, poor water solubility, and nonspecific drug delivery at the target site [10]. To address these issues, various groups have studied the modes of action of traditional medicinal plants. The analysis of the mechanism of action from various studies concludes that the medicinal plants mostly target the apoptotic pathways, both intrinsic and extrinsic, to show their cytotoxicity in cancer cells. Most studies have focused on breast and cervical cancers. The anticancer activities of *A. pilosa*, *A. officinarum*, *A. muricata*, *A. cinnamomea*, *A. hamosus*, and *A. indica* are mediated by the modulation of multiple key signaling pathways across different cancer types, including fibrosarcoma, breast, gastric, esophageal, neuroblastoma, colorectal, lung, cervical, and liver cancers. *A. pilosa* attenuates the phosphorylation of ERK, JNK, and AKT1 in fibrosarcoma cells, downregulates MMP‐2 and MMP‐9 expression, and inhibits ROS accumulation, resulting in reduced invasion and metastatic potential. In breast cancer, *A. officinarum* and its flavonoid galangin reduce the proliferation of breast cancer cells by downregulating S‐phase cyclins (E2F1, CDK2, and cyclin A) and inducing apoptosis via mitochondrial and caspase‐dependent pathways. Galangin inhibits key inflammatory cytokines, such as IL‐6, IL‐1*β*, and TNF‐*α*, by targeting COX‐2 and disrupting the JAK/STAT3 signaling pathway to impair cell survival in gastric cancer. In neuroblastoma, it upregulates apoptosis by altering the BCL‐2/Bax ratio and Caspase‐3 activation. *A. muricata* exhibits broad‐spectrum anticancer effects in colon, breast, and prostate cancer models by downregulating key signaling pathways, namely MAPK, EGFR/PI3K/AKT, and NF‐*κ*B. It promotes G1‐phase cell cycle arrest, ROS generation, mitochondrial dysfunction, cytochrome c release, and downregulation of BCL‐2. In colorectal cancer, the extracts disrupt crucial metabolic processes, including glycolysis, the urea cycle, and amino acid metabolism, suppressing cancer cell proliferation and migration. Similarly, *A. cinnamomea* exerts anticancer activity in lung tamoxifen‐resistant breast cancer by upregulating Tp53 and miRNAs (miR 21/26/30), inducing S‐phase arrest, downregulating SKP2, and inhibiting MMP‐2/9 to prevent metastasis. In liver cancer (HCC), its extract improves liver function, enhances Bax, and reduces alpha‐fetoprotein levels. *A. hamosus* exhibits cytotoxicity in ER+ breast cancer by inducing apoptosis through Caspase‐3/8/9 activation, BCL‐2 and Ki67 downregulation, and cell cycle arrest, while modulating immune responses in other cancers, such as lung, colon, and prostate cancers. *A. indica* (neem) exhibits broad‐spectrum anticancer activity in breast, cervical, and colon cancers by suppressing NF‐*κ*B, inducing mitochondrial apoptosis via Bax/Caspase‐3/9 activation, and inhibiting PI3K/AKT/mTOR and cyclin‐CDK complexes. Its bioactive compound, nimbolide, further enhances apoptosis and reduces metastasis in xenograft models. *B. alba* exerts cytotoxic effects against EAC by triggering both intrinsic and extrinsic apoptotic pathways. This involves an increase in Fas, Bax, Caspases‐3, ‐8, ‐9, cytochrome c, and Tp53, along with the regulation of NF‐*κ*B and PARP‐1 activities [56]. *C. procera* exhibits cytotoxic activity against breast, glioblastoma, and prostate cancers by inhibiting the PI3K/AKT/mTOR pathway, scavenging of ROS, apoptosis activation, and ERK1/2 downregulation, ultimately suppressing angiogenesis and metastasis [17]. *C. sinensis* (green tea), particularly its polyphenol EGCG, exhibits antiproliferative and proapoptotic effects in different cancer types, including breast, colon, and prostate cancers, by affecting the NF‐*κ*B, VEGF, HGF, MMP‐9, IL‐23, and caspase pathways [59, 60, 62]. It also downregulates endocrine‐related growth factors, making it effective in hormone‐independent breast cancer models. *C. colocynthis* exhibits anticancer effects in colon and lung cancers through its bioactive cucurbitacins, which interfere with the cell cycle, leading to a reduction in cell proliferation and inducing apoptosis, possibly by downregulating the antiapoptotic protein (BCL‐2) and modulating cytokines and oxidative stress [22]. *E. elliptilimba* exerts antiproliferative activity against HER2+ and breast cancer cells, inducing G2/M cell cycle arrest, likely by interacting with tubulin and disrupting mitotic progression [63]. *E. billardieri* exhibits anticancer effects in pancreatic cancer by activating the intrinsic apoptotic pathway and inhibiting cell proliferation. Its DCM and n‐hexane extract upregulated Bax, promoting apoptosis with minimal necrosis, and downregulated cyclin D1, leading to cell cycle arrest. In addition, these effects are selective to cancerous cells, suggesting their potential as therapeutic agents [66]. *Gossypol, derived from G. hirsutum*, induces apoptosis in breast cancer and colon cells by inhibiting BCL‐2 at both transcriptional and protein levels and affecting metabolic genes related to inflammation, lipid biosynthesis, and glucose metabolism [141, 142]. Lastly, *G. speciosum* shows promising antitumor effects in breast cancer cells, reducing AKT/*β*‐catenin signaling and inhibiting proliferation and colony formation through its active component, isovitexin, highlighting its potential as a tumor‐static agent [70]. Several extracts modulate critical oncogenic pathways, such as PI3K/AKT/mTOR (e.g., *P. notoginseng, C. procera*), Wnt/*β*‐catenin (e.g., *G. speciosum*, *V. myrtillus*), JAK/STAT (e.g., *L. speciosa*), and NF‐*κ*B (e.g., *T. diffusa*). Notably, *J. adhatoda* induces apoptosis and autophagy in breast and colorectal cancer cells by downregulating mTOR and triggering Beclin‐1, LC3, and caspases. *P. palatiferum* triggers ER‐stress‐mediated cell death via Ca^2+^ overload and oxidative stress. Compounds such as corosolic acid, ginsenosides, gossypol, and phenanthroindolizidine alkaloids contribute to these effects by modulating proapoptotic proteins (Fas, Bax, and caspases) and suppressing antiapoptotic factors (survivin and BCL‐2). Collectively, these findings underscore the therapeutic potential of phytochemicals in targeting specific cancers through defined molecular pathways.

**Nomenclature**
2‐DAH: 2‐diarylheptanoids
5‐HHMPH: 5‐hydroxy‐7‐(4″‐hydroxy‐3″‐methoxyphenyl)‐1‐phenyl‐3‐heptanone
AA: asiatic acid
ACE: *Antrodia cinnamomea* extract
AG: aqueous green
AKT 1: protein kinase B
AM: *Annona muricata*
AMEA: *Annona muricata* ethyl acetate extract
AMCE: *Annona muricata* chloroform extract
AP: *Agrimonia pilosa*
Atg5: autophagy‐related protein 5
Atg12: autophagy‐related protein 12
BA: betulinic acid
BAX: associated X protein (proapoptotic)
BaME: *Basella alba* methanolic extract
BCL2: B‐cell lymphoma 2 (antiapoptotic)
Beclin‐1: autophagy‐related protein
Bid: BH3‐interacting domain death agonist
BNIP3‐: Bcl‐2/adenovirus E1B 19 kDa interacting protein 3
CNA: carnosic acid
CNS: central nervous system
CRA: corosolic acid
CS: carnosol
CDDP: cisplatin
CDK1: cyclin‐dependent kinase 1
CDK2: cyclin‐dependent kinase 2
CENPF: centromere protein F
CPT: camptothecin
COX‐2: Cyclooxygenase‐2
Cyt‐C: cytochrome c
CYP1A1: Cytochrome P450 Family 1 Subfamily A Member 1
CUR: Curcumin
CRISPR/Cas9: Clustered Regularly Interspaced Short Palindromic Repeats/CRISPR‐associated protein 9
DCM: Dichloromethane extract
DDX3: DEAD‐box helicase 3
DMBA‐7,12: dimethylbenz[a]anthracene
EAC: Ehrlich ascites carcinoma
EAD: epoxyazadiradione
ECG: epicatechin‐3‐gallate
EG: epigallocatechin
EGCG: epigallocatechin‐3‐gallate
EGFR: epidermal growth factor receptor
ELISA: enzyme‐linked immunosorbent assay
ER+: estrogen receptor positive
ER‐stress: endoplasmic reticulum Stress
ERK: extracellular signal‐regulated kinase
Fas: Fas receptor (CD95)
FasL: Fas ligand
GSE: *Grammatophyllum speciosum* ethanolic extract
GSH: glutathione
HCC: hepatocellular carcinoma
HER2+: human epidermal growth factor receptor 2 positive
HGF: hepatocyte growth factor
IF: Apoptosis‐inducing factor
IL‐1*β*, IL‐6, IL‐23: Interleukins / inflammatory cytokines
JAK2: Janus kinase 2
JNK‐c: Jun N‐terminal kinase
Ki67: marker of proliferation
KFD: Kaempferide
LC3: microtubule‐associated protein 1A/1B‐light chain 3
LC3‐I: cytosolic form of LC3
LC3‐II: lipidated autophagosome‐associated form of LC3
LC‐MS/MS: liquid chromatography–tandem mass spectrometry
LH: Luteinizing Hormone
LPS: Lipopolysaccharide
MAPK: mitogen‐activated protein kinase
MMP/MMP‐2, MMP‐9: matrix metalloproteinases
mTOR: mammalian target of rapamycin
MNBE: methanolic Neem bark extract
NF‐*κ*B: nuclear factor kappa B
NO: nitric oxide
NOD‐SCID: nonobese diabetic severe combined immunodeficient
OA: oleanolic acid
PARP: poly (ADP‐ribose) polymerase
PBMCs: peripheral blood mononuclear cells
PCNA: proliferating Cell Nuclear Antigen
PDK1: 3‐phosphoinositide‐dependent protein kinase‐1
PI3K: Phosphoinositide 3‐kinase
PR+: Progesterone receptor positive
PNS: *Panax notoginseng* saponins
PTEN: phosphatase and tensin homolog
R7: notoginsenoside R7
Rg3: ginsenoside Rg3
Rh2: ginsenoside Rh2
Raptor: regulatory‐associated protein of mTOR
RA: rosmarinic acid
ROS: reactive oxygen species
SQSTM1/p62: Sequestosome‐1
STAT3: signal transducer and activator of transcription 3
TB‐AG: *Terminalia brownii*‐derived green silver nanoparticles
TNBC: triple‐negative breast cancer
TNF: *α*‐tumor necrosis factor alpha
Tp53: tumor protein p53
TRAIL: TNF‐related apoptosis‐inducing ligand
VEGF: vascular endothelial growth factor
VEGFR2: vascular endothelial growth factor receptor 2
Wnt: wingless/Integrated

## Author Contributions

Dr. Suparna Laha contributed to the conceptualization, original draft, study design, funding acquisition, supervision, visualization, and manuscript review and editing. Ms. Mithila Kulkarni was responsible for the original draft preparation, data curation, formal analysis, investigation, literature review, editing, and figure preparation. Mr. Amjad Hussain contributed to the manuscript review, editing, data curation, formal analysis, investigation, and preparation of figures and tables. Mr. Reginald Samson Valdar contributed to the manuscript review and referencing, investigation, and assisted in the literature review. Ms. Shreya Hebbar partially contributed to the original draft and literature review.

## Funding

This study was supported by Yenepoya University (10.13039/100023000, YU/Seed grant/132‐2022).

## Ethics Statement

The authors have nothing to report.

## Consent

The authors have nothing to report.

## Conflicts of Interest

The authors declare no conflicts of interest.

## Data Availability

Data sharing is not applicable to this article as no datasets were generated or analyzed during the current study.
